# Foxf2 and Smad6 co‐regulation of collagen 5A2 transcription is involved in the pathogenesis of intrauterine adhesion

**DOI:** 10.1111/jcmm.14708

**Published:** 2020-02-05

**Authors:** Guobin Chen, Limin Liu, Jing Sun, Liying Zeng, Huihua Cai, Yuanli He

**Affiliations:** ^1^ Department of Obstetrics and Gynecology Zhujiang Hospital Southern Medical University Guangzhou China; ^2^ Department of Obstetrics and Gynecology Shenzhen Maternity and Childcare Hospital Shenzhen China; ^3^ Department of Obstetrics and Gynecology Guangdong Provincial People`s Hospital, Guangdong Academy of Medical Sciences Guangzhou China

**Keywords:** fibrosis, Foxf2, intrauterine adhesion, Smad6

## Abstract

The replacement of normal endometrial epithelium by fibrotic tissue is the pathological feature of intrauterine adhesion (IUA), which is caused by trauma to the basal layer of the endometrium. COL5A2 is a molecular subtype of collagen V that regulates collagen production in fibrotic tissue. Here, we investigated the roles of Foxf2 and Smad6 in regulating the transcription of COL5A2 and their involvement in the pathogenesis of IUA. Small interference‐mediated Foxf2 (si‐Foxf2) silencing and pcDNA3.1‐mediated Smad6 (pcDNA3.1‐Smad6) up‐regulation were performed in a TGF‐β1‐induced human endometrial stromal cell line (HESC) fibrosis model. Assessment of collagen expression by Western blotting, immunofluorescence and qRT‐PCR showed that COL5A2, COL1A1 and FN were significantly down‐regulated in response to si‐Foxf2 and pcDNA3.1‐Smad6. Transfection of lentivirus vector‐Foxf2 (LV‐Foxf2) and pcDNA3.1‐Smad6 into HESCs and qRT‐PCR showed that Foxf2 promoted COL5A2 expression and Smad6 inhibited Foxf2‐induced COL5A2 expression. Co‐immunoprecipitation, chromatin immunoprecipitation and dual‐luciferase reporter assays to detect the interaction between Foxf2 and Smad6 and their role in COL5A2 transcription showed that Foxf2 interacted with Smad6 and bond the same promoter region of COL5A2. In a rat IUA model, injection of ADV2‐Foxf2‐1810 and ADV4‐Smad6 into the uterine wall showed that Foxf2 down‐regulation and Smad6 up‐regulation decreased fibrosis and the expression of COL5A2 and COL1A1, as detected by haematoxylin/eosin, Masson trichrome staining and immunohistochemistry. Taken together, these results suggested that Foxf2 interacted with Smad6 and co‐regulated COL5A2 transcription in the pathogenesis of IUA, whereas they played opposite roles in fibrosis.

## 1 INTRODUCTION

Intrauterine adhesion (IUA) is a disease caused by injury to the basal layer of the endometrium resulting in partial or complete obliteration of the uterine cavity and/or the cervical canal. IUA is a major health problem involving the female reproductive system for women of childbearing age. It can lead to menstrual abnormalities, periodic abdominal pain, recurrent abortion, infertility and pregnancy‐related complications, such as placenta adhesion and placenta accrete.^1^ Most cases of IUA occur after dilation and curettage for missed abortion, selective termination of pregnancy and postpartum placental residual.^2^, ^3^ The pathogenesis of IUA involves decreased or absent endometrial glands, and the endometrial stroma is mostly replaced by fibrous tissue, leading to uterine cavity deformation and endometrial fibrosis.^4^ Biopsy samples from the uterine wall of patients with IUA contain 50%‐80% of fibrous tissue, compared with 13%‐20% in patients without IUA.^5^

**Figure 1:**
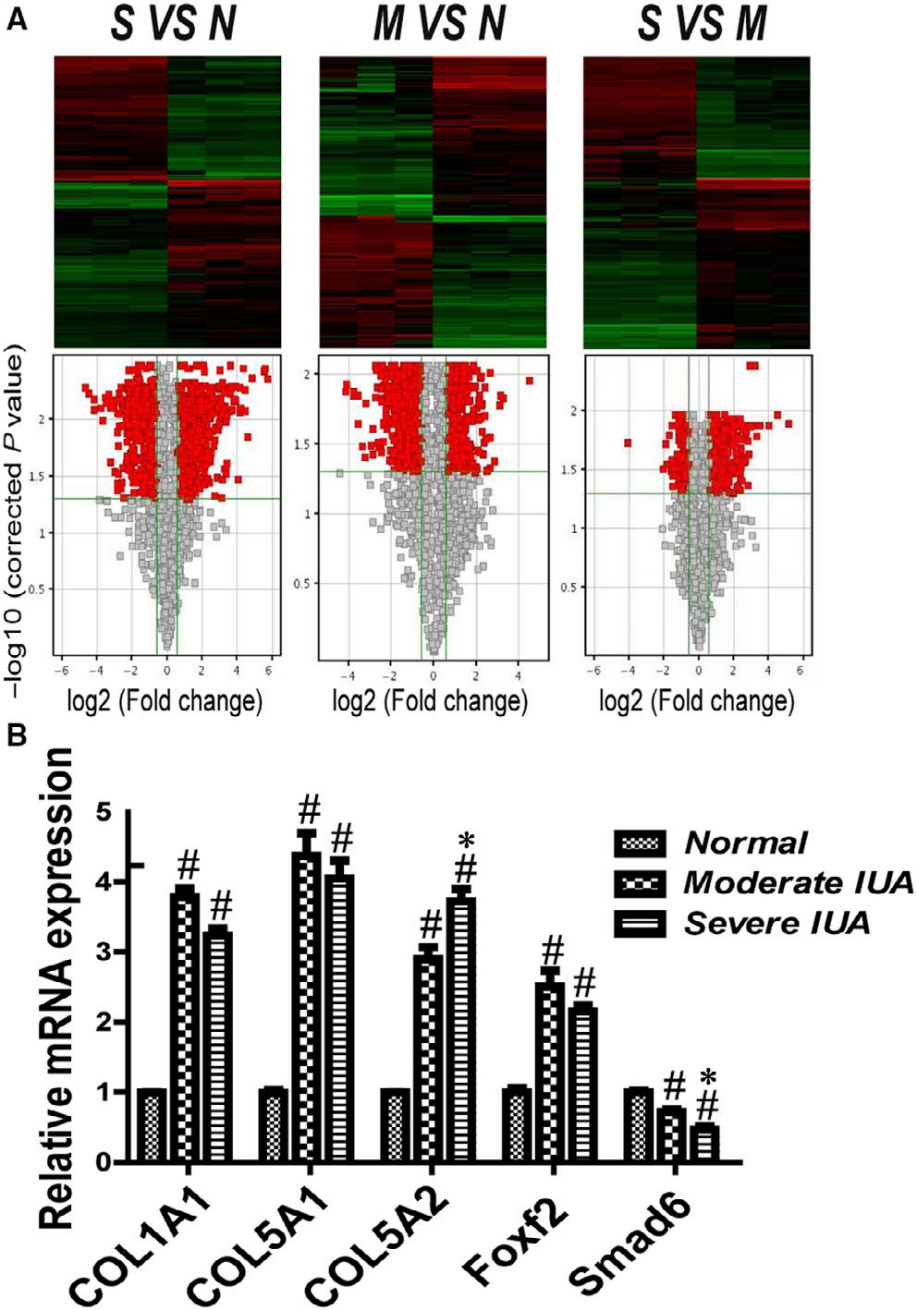
Microarray sequencing for gene expression profiles of endometrial specimen (n = 5). (A) Heat map and Volcano plot representation of tissue microarray sequencing for gene expression profiles of IUA and normal control. Abbreviations: M, moderate IUA group; N, normal group; S, severe IUA group. (B) The relative mRNA expression of COL1A1, COL5A1, COL5A2, Foxf2 and Smad6 in each group. ^#^ *P* < .05, compared with normal group. *P < .05, compared with moderate IUA group

Excessive deposition of extracellular matrix (ECM) substituting the normal endometrium is the characteristic feature of endometrial fibrosis.^6^ Collagen is the major component of the ECM and plays a vital role in wound healing; however, excessive collagen production leads to organ fibrosis.^7^, ^8^ More than 20 types of collagen have been found, and the most abundant subtypes are types I, III and V, which expressed extensively in fibrous tissue.^9^ In our unpublished study, we collected 15 endometrial specimens including five normal, five moderate IUA and five severe IUA samples, which were used for microarray sequencing for gene expression profiles. The results showed that COL5A1, COL5A2 and COL1A1 were expressed at higher levels in the IUA group than in the normal group, especially COL5A2, which was correlated with the degree of IUA (Figure 1A,B).

Type V collagen (COLV) is a regulatory fibril‐forming collagen that plays an important role in the formation of fibrils; it acts in combination with collagens I and III in the formation of fibrils and their deposition in the ECM.^10^ COLV includes three different isoforms, COL5A1, COL5A2 and COL5A3.^11^ Abnormal expression of COL5A2 is associated with many fibrous diseases, and COL5A2 expression is increased in tubulointerstitial fibrosis and systemic sclerosis.^12^, ^13^ Based on these findings together with the results of our previous study, we have been suggested that COL5A2 might play a vital role in the pathogenesis of IUA. Therefore, we examined the mechanism underlying the regulation of COL5A2 and its role in the pathogenesis of IUA. Forkhead box F2 (Foxf2) is a transcription factor that is widely expressed in mesenchymal tissues and plays an important role in organ development and ECM formation.^14^, ^15^ Collagens within the ECM are dramatically decreased in the intestines of Foxf2 knockout mice, resulting in tissue disintegration.^16^ This suggests that Foxf2 is important for collagen production. The TGF‐β/Smad signalling pathway is involved in the pathogenesis of IUA, as shown in vivo and in vitro.^17^, ^18^ In our previous study, we demonstrated the involvement of the TGF‐β/Smad signalling pathway in the fibrosis of primary human endometrial stromal cells.^19^ Smad6 is the downstream mediator of the TGF‐β superfamily and can negatively regulate the TGF‐β signalling pathway.^20^ Overexpression of Smad6 inhibits collagen production by suppressing epithelial‐mesenchymal transition (EMT).^21^ In conjunctival fibrosis, valproic acid decreases collagen expression by up‐regulating the expression of Smad6.^22^

Both Foxf2 and Smad6 are related to the production of collagen, and Foxf2 and Smad6 expression were correlated with the degree of IUA (Figure 1B). Therefore, we speculated that Foxf2 and Smad6 are important for the pathogenesis of IUA. Both Foxf2 and Smad6 are transcription factors; however, whether they are correlated with the expression of COL5A2 has not been reported to date. We predicted the presence of transcription factor binding sites for Foxf2 or Smad6 in the promoter region of COL5A2 that could be involved in the regulation of its transcription (http://jaspar.genereg.net/, http://genome.ucsc.edu/).

In the present study, small interference‐mediated Foxf2 silencing (si‐Foxf2) and pcDNA3.1‐mediated Smad6 up‐regulation (pcDNA3.1‐Smad6) were transfected into a TGF‐β1‐induced HESC fibrosis model in vitro. Western blotting, immunofluorescence (IF) staining and qRT‐PCR were used to measure the expression of COL5A2, COL1A1 and FN. The EdU assay and flow cytometry were performed to examine cell proliferation and cycle progression. Co‐immunoprecipitation (Co‐IP), chromatin immunoprecipitation (ChiP) and dual‐luciferase reporter assays were performed to confirm the interaction between Foxf2 and Smad6 and their regulation of COL5A2 transcription. Foxf2 was down‐regulated using ADV2‐Foxf2‐1810, and Smad6 was up‐regulated using ADV4‐Smad6 in a female SD rat IUA model in vivo. Haematoxylin and eosin (HE) and Masson trichrome immunostaining were used to examine the glands and the fibrosis area in the endometrium. Immunohistochemistry (IHC) was performed to detect the expression of COL5A2 and COL1A1. We showed that Foxf2 down‐regulation and Smad6 up‐regulation inhibited fibrosis in vivo and in vitro. Foxf2 and Smad6 play opposite roles in fibrosis by binding to the same promoter region of COL5A2 and regulating its transcription, thereby affecting the pathogenesis of IUA.

## 2 MATERIALS AND METHODS

### 2.1 Cell culture and group

Human endometrial stromal cell lines (HESCs) were purchased from ATCC (Manassas, USA). Cells were cultured in 1:1 mixture of Dulbecco's modified Eagle's medium and Ham's F12 medium (DMEM/F12, Gibco, USA) without phenol red, supplemented with 1 mmol/L sodium pyruvate (HyClone, USA), 1.5 g/L sodium bicarbonate (Leagene, Beijing, China), 1% ITS+ Premix (Corning, USA), 500 ng/mL puromycin, 10% foetal bovine serum (Gibco, USA), 100 IU/mL penicillin and 0.1 mg/mL streptomycin at 37°C in a 5% CO_2_ incubator. For cell fibrosis model, the cells were stimulated with TGF‐β1 (PeproTech, USA). The study was divided into nine groups: normal group (without any intervention), TGF‐β1 stimulation group, si‐negative control group (TGF‐β1 stimulation and transfection with si‐Foxf2‐negative control), si‐Foxf2‐1415 group (TGF‐β1 stimulation and transfection with si‐Foxf2‐1415), si‐Foxf2‐650 group (TGF‐β1 stimulation and transfection with si‐Foxf2‐650), pcDNA3.1 group (TGF‐β1 stimulation and transfection with pcDNA3.1), pcDNA3.1‐Smad6 group (TGF‐β1 stimulation and transfection with pcDNA3.1‐Smad6), si‐Foxf2‐1415+ pcDNA3.1‐Smad6 group (TGF‐β1 stimulation and transfection with pcDNA3.1‐Smad6 and si‐Foxf2‐1415) and si‐Foxf2‐650+ pcDNA3.1‐Smad6 group (TGF‐β1 stimulation and transfection with pcDNA3.1‐Smad6 and si‐Foxf2‐650).

### 2.2 si‐Foxf2 and pcDNA3.1‐Smad6 and recombinant adenovirus constructs and transfection

Small interference‐mediated Foxf2 (si‐Foxf2) silencing and its negative control were purchased from GenePharma (Shanghai, China). The sequences of si‐Foxf2‐1415, Si‐Foxf2‐650 and negative control are shown in Table 1. pcDNA3.1‐Smad6 was constructed by inserting an open reading frame of Smad6 into pcDNA3.1 for the purpose of up‐regulating Smad6 expression (GenePharma). ADV2‐Foxf2‐1810 and ADV4‐Smad6 (rat) were purchased from GenePharma, and the sequences are shown in Table 1. Lipofectamine 3000 (Invitrogen) was used to transfect si‐Foxf2 and pcDNA3.1‐Smad6 into HESCs. Because the efficiency of si‐Foxf2 in vivo was low in our pre‐experiment, we chose ADV2‐Foxf2‐1810 to down‐regulate Foxf2 expression and ADV4‐Smad6 to up‐regulate Smad6 expression in rat IUA model. ADV2‐Foxf2‐1810 and ADV4‐Smad6 were directly injected into rat uterine wall. All the transfection procedures were according to the protocol of the manufacturer.

**Table 1:** The sequence of si‐Foxf2 and ADV2‐Foxf2‐1810 and their negative control

	Sense (5′－3′)	Antisense (5′－3′)
si‐Foxf2‐1415	GCGUCUGUC AGGAUAUUAATT	UUAAUAUCCUGACAGACGCTT
si‐Foxf2‐650	CCAGCGAGUUCAUGUUCGATT	UCGAACAUGAACUCGCUGGTT
Negative control	UUCUCCG AACGUGUCACGUTT	ACGUGACACGUUCGGAGAATT
ADV2‐Foxf2‐1810	GGCGACAACTTCCATCATT	
ADV2‐NC	TTCTCCGAACGTGTCACGTTTC	

### 2.3 RNA extraction, reverse transcription and quantitative real‐time PCR (qRT‐PCR)

TRIzol reagent (Takara, Japan) was used to extract total RNAs from HESCs or tissues. PrimeScript™RT Reagent Kit with gDNA Eraser (Takara) was used to perform reverse transcription. Quantitative real‐time PCR was performed by using the CFX Connect Real‐Time System (Bio‐Rad, USA) with the SYBR Green Kit (Takara) according to the manufacturer's instruction. The primer sequences were synthesized by Sangon Biotech (Shanghai, China) as listed in Table 2. Calculation of the targeted mRNAs was based on the Cq results and normalization to GAPDH expression. All of the reactions were performed in triplicate.

**Table 2:** The sequence of primer for quantitative real‐time PCR

	Gene	Forward primer sequence (5′－3′)	Reward primer sequence (5′－3′)
Human	Foxf2	TCGCTGGAGCAGAGCTACTT	CCCATTGAAGTTGAGGACGA
	Smad6	AGACGGCGTTGGCCTTT	CCTGCCTTTACCTTGCCTTTT
	COL5A2	TCTTGCTCCTGTGGATGTTG	TTGATGGTGGTGCTCATTGT
	COL1A1	GAGGGCCAAGACGAAGACATC	CAGATCACGTCATCGCACAAAC
	FN	ACAACCCCTACAAACGGCCA	TAGTCAATGCCCGGCTCCAG
Rat	GAPDH	GCGGGGCTCTCCAGAACATCAT	GACGCCTGCTTCACCACCTTCTT
	Foxf2	GACTACTTGCACCAGAACGCC	ACACGCTCTGGTGZTGG
	Smad6	CCTATTCTCGGCTGTCTCCTCCTG	GGCTTGGCTTGGCATCTG
	GAPDH	GGTGGACCTCATGGCCTACA	CTCTCTTGCTCTCAGTATCCTTGCT

### 2.4 Proteins extraction and Western blotting

Total proteins were extracted from HESCs with RIPA lysis buffer (Cwbiotech, Beijing, China). The protein concentrations were measured by BCA reagent kit (Merck, USA). The proteins were resolved by SDS‐PAGE (Beyotime Biotechnology, Shanghai, China) and then transferred to PVDF membrane (Millipore, USA), incubated with rabbit anti‐Foxf2 monoclonal antibody (1:1000, Abcam, USA), rabbit anti‐Smad6 polyclonal antibody (1:1000, Abcam, USA), rabbit anti‐COL1A1 polyclonal antibody (1:1000, Abcam, USA), rabbit anti‐fibronectin polyclonal antibody (1:1000, Abcam, USA), mouse anti‐COL5A2 polyclonal antibody (1:200, Santa Cruz Biotechnology, USA) overnight at 4°C, following incubated with anti‐rabbit or antimouse IgG (H+L) biotinylated antibody (CST, USA), developed in ChemiDoc™ XRS+Imaging System (Bio‐Rad) using the chemiluminescence method (ECL, Millipore, USA), normalized with GAPDH (CST, USA).

### 2.5 Cell proliferation assay

EdU assay was used to detect cell proliferation according to the manufacturer's instructions (Cwbiotech). The cells transfected with si‐Foxf2 or (and) pcDNA3.1‐Smad6 were cultured in 96‐well plates at a density of 1 × 10^4^ cells/well. Forty‐eight hours after TGF‐β1 stimulation, the cells were incubated with 50 μmol/L EdU at 37°C in a 5% CO_2_ incubator for 2 hours, and then fixed with 4% paraformaldehyde. All the experimental procedure was referred to as protocol.^23^ The nucleus was stained with Hoechst 33342 for 30 minutes. Cell images were captured with an inverted fluorescence microscope (Leica, Germany). The proliferation rate was defined as the percentage of EdU‐positive cells.

### 2.6 Flow cytometric analysis

For cell cycle analysis, forty‐eight hours after transfection with si‐Foxf2 or (and) pcDNA3.1‐Smad6, the cells were fixed in 70% ethanol at least 2 hours at 4°C, supplemented with RNaseA and propidium iodide (KeyGEN, China) for 30 minutes in the dark. The percentage of cells at G0/G1, S and G2/M phases were analysed by flow cytometer (BD FACS Verse; BD, USA). All the reactions were performed in triplicate.

### 2.7 Immunofluorescent staining (IF)

Seventy‐two hours after transfection, the cells were fixed in 4% paraformaldehyde for 15 minutes, and then in 0.1% Triton X‐100 for 15 minutes, blocked in 5% bovine serum albumin (BSA) for 2 hours, incubated with mouse anti‐COL5A2 polyclonal antibody (1:50, Santa Cruz Biotechnology, USA), rabbit anti‐Foxf2 monoclonal antibody (1:200, Abcam, USA), rabbit anti‐Smad6 polyclonal antibody (1:200, Abcam, USA) overnight at 4°C, followed by secondary antibodies against rabbit and mouse (Thermo, USA), and then counterstained with 4′, 6‐diamidino‐2‐phenylindole (DAPI) for 15 minutes. Images were captured by laser confocal microscopy system (Carl Zeiss, Jena, Germany) at a magnification of 630×.

### 2.8 Co‐immunoprecipitation (Co‐IP) assay

HESCs were harvested after stimulation of TGF‐β1 for 72 hours. Catch and Release® v2.0 Reversible Immunoprecipitation System (Millipore, USA) was used for Co‐IP test. The procedure followed the manufacturer's instruction. The cells were lysed, and then, 2 μg/mL of antibodies (Foxf2 or Smad6) was used to precipitate proteins. Anti‐rabbit immunoglobulin G monoclonal antibody (1:1000, Sigma‐Aldrich) was used as negative control in the experiments. The precipitated proteins were resolved to SDS‐PAGE and transferred to a PVDF membrane. The membranes were incubated with primary antibodies against Foxf2 or Smad6 (Abcam) overnight at 4°C, followed with secondary anti‐rabbit horseradish peroxidase‐conjugated IgG (1:1000, CST, USA), developed in ChemiDoc™ XRS+Imaging System using the chemiluminescence method (ECL, Millipore).

### 2.9 Chromatin immunoprecipitation (ChIP)

We predicted three binding sites at the promoter regions of COL5A2 that Foxf2 or Smad6 may have the potential to bind (http://jaspar.genereg.net/, http://genome.ucsc.edu/). HESCs were stimulated with TGF‐β1 for 72 hours and then cross‐linked in 1% formaldehyde for 10 minutes at 37°C. ChIP assay was performed using EZ ChIP kit (Millipore, USA) according to the manufacturer's instruction. The antibodies against Foxf2 or Smad6 (Abcam, USA) were used to precipitate chromatin DNA, with a normal lgG as a control. And then, the DNA was retrieved and purified. qPCR was performed to confirm the binding sites at chr2:190046519‐190046820, chr2:190067994‐190068233 and chr2:190069038‐190069375 where Foxf2 or Smad6 may bind, following the protocol: pre‐denaturation at 95°C for 10 minutes, denaturation at 95°C for 15 seconds and extension 60°C for 60 seconds. The sequence of primers for predicted binding sites is listed in Table 3.

**Table 3:** The sequence of primers for predicted binding sites for quantitative real‐time PCR

Gene		Sequence (5′－3′)
Domain 1	F	GTTAATAAAGTTGTTTTAAATTTAACTATAAACATGGCT
	R	GTCATACCATGAGCTTTCAGTAGGG
Domain 2	F	TGCTTTGGCAGATGTGGAGT
	R	GCCGATACATTGCAACTTTGG
Domain 3	F	GTAATCTTAAATTGTCTTACATACACTTTCGAAC
	R	ATCTAAAGGAAAAATGAATTAAAGGAGAGAG
GAPDH Promoter	F	CATGGGTGTGAACCATGAGA
	R	GTCTTCTGGGTGGCAGTGAT

### 2.10 Dual‐luciferase reporter assay

To detect which binding site where Foxf2 and Smad6 may bind at the promoter region of COL5A2, we synthesized three binding site sequences by chemosynthesis and amplified them by PCR. The PCR products were inserted into PGL3‐basic vector, respectively, PGL3‐COL5A2‐1, PGL3‐COL5A2‐2 and PGL3‐COL5A2‐3 (GenePharma). Wild‐type and mutant Foxf2 and Smad6 plasmids were synthesized by GenePharma. The wild‐type and mutant Foxf2 or Smad6 recombinant plasmids were transfected with PGL3‐COL5A2‐1, PGL3‐COL5A2‐2 and PGL3‐COL5A2‐3, respectively, in 293T cells for 48 hours. Dual‐Luciferase Reporter Assay System (Promega, Madison, USA) was used to measure luciferase. Renilla luciferase was used as a control. The experiments were performed in triplicate.

### 2.11 Experimental animals and the creation of a rat IUA model

Experiment protocols were approved by Animal Care and Use Committees of Southern Medical University. Adult female SD rats weighing 200～250 g were purchased from the Animal Center of Southern Medical University (Guangzhou, China). The rats were fed in standardized laboratory conditions in a temperature‐controlled room and light conditions (12‐hour light and 12‐hour dark) with standard chow and water ad libitum. In our previous study, we had established the IUA model by curettage and infection (dual damage) to the endometrium.^24^ In this study, we still exerted dual damage to female rat endometrium to create rat IUA model. The animals were randomly divided into six groups: sham operation, IUA group (dual damage to create IUA), ADV‐negative control group (dual damage to create IUA and transfect with ADV‐negative control), Foxf2 down‐regulation therapy group (dual damage to create IUA and transfect with ADV2‐Foxf2‐1810), Smad6 up‐regulation therapy group (dual damage to create IUA and transfect with ADV4‐Smad6) and combination therapy group (dual damage to create IUA and cotransfect with ADV2‐Foxf2‐1810 and ADV4‐Smad6). Each group had five rats (n = 10 uterine horns). When creating the IUA model, the rat uterine wall was injected with ADV2‐Foxf2‐1810 or (and) ADV4‐Smad6. The uterine cavity appearance is nodular if the creation of IUA model was success, and adhesion can be observed with naked eye when the horn opened.

### 2.12 Evaluation of Adhesion Severity

In our previous study, we demonstrated that the time for the formation of IUA model was two weeks optimally.^24^ Thus, in this study, the rats were killed by injection of urethane two weeks after creation of IUA model. The uteri were immediately excised, fixed in 4% paraformaldehyde, embedded in paraffin and sliced. Haematoxylin‐eosin and Masson trichrome staining were performed. The number of endometrial glands and the percentage of endometrial fibrosis area were evaluated in a microscope as described previously.^24^ The observer was blind to each group.

### 2.13 Immunohistochemistry

The above paraffin sections were deparaffinized, rehydrated, antigen‐retrieved by microwave heat for 20 minutes, and then immersed in 3% hydrogen peroxide for 15 minutes to block endogenous peroxidase activity and blocked in 5% bovine serum albumin for 10 minutes at 37°C. The slides were incubated with anti‐Foxf2 antibody, anti‐Smad6 antibody, anti‐COL1A1 antibody (1:100 dilution, Abcam, USA) and anti‐COL5A2 antibody (1:200 dilution, Abnova, Taiwan) overnight at 4°C, followed by incubation with secondary antibody (SignalStain® Boost IHC Detection Reagent; CST, USA) 1 hour, stained with 3,3‐diaminobenzidine (DAB), counterstained with haematoxylin (Solarbio, China), and dehydrated and mounted as described previously.^25^ All slides were observed by a blind investigator under a microscope (400×). The images of COL5A2, COL1A1, Foxf2 and Smad6 were captured by microphotography. The staining was evaluated by semi‐quantitatively scored using the modified histochemical score (H‐score). Staining intensity was categorized four degrees: 0 for negative, 1+ for weak staining, 2+ for moderate staining and 3+ for strong staining. The percentage of different staining intensity cells was determined by visual assessment. And then, the score was calculated using the formula 1 × (% of 1+ cells) + 2 × (% of 2+ cells) + 3 × (% of 3+ cells) and produced a final score ranging from 0 to 300.^26^ All cases were scored without prior knowledge of each group.

### 2.14 Statistical analysis

Statistical analysis was performed using the SPSS 20.0 software (SPSS Inc, Chicago, IL, USA). Data were expressed as the mean ± standard deviation. Comparisons of the measurement data were performed by the t test and categorical data by the chi‐square test. A *P* < .05 was considered to be statistically significant.

## 3 RESULTS

### 3.1 Establishment of a HESC fibrosis model

**Figure 2:**
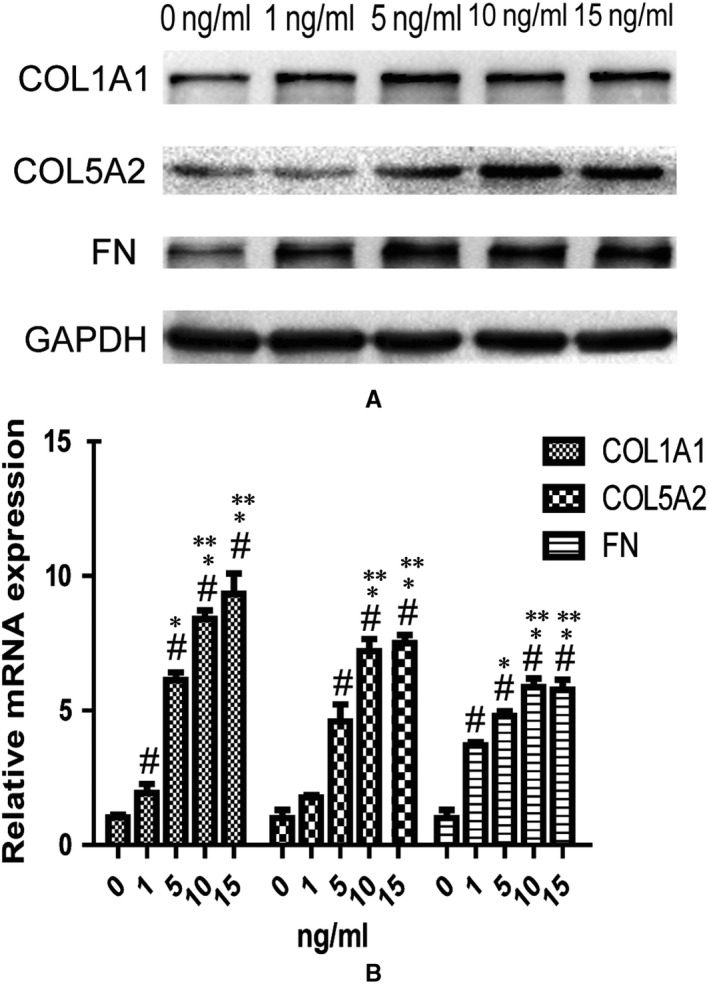
Human endometrial stromal cell lines (HESCs) were treated with TGF‐β1 (0, 1, 5, 10, 15 ng/mL) to select an optimal dosage to create a cell fibrosis model (n = 3). (A) Western blotting analysis of COL1A1, COL5A2 and FN in different concentration groups of TGF‐β1. (B) qRT‐PCR analysis of the mRNA expressions of COL1A1, COL5A2 and FN in different concentration groups of TGF‐β1. ^#^ *P* < .05, compared with control group (0 ng/mL). *P < .05, compared with 1 ng/mL group. **P < .05, compared with 5 ng/mL group

The TGF‐β signalling pathway plays a pivotal role in the development of fibrosis, and TGF‐β1 is an important pro‐fibrotic cytokine.^27^ In our previous study, we used TGF‐β1 to stimulate primary human endometrial stromal cells to generate a cell fibrosis model. The results showed that 10 ng/mL TGF‐β1 was the optimal concentration to stimulate fibrosis in primary human endometrial stromal cells.^21^ In the present study, we used TGF‐β1 to create a HESC fibrosis model. Cells were incubated with different concentrations of TGF‐β1 (0, 1, 5, 10 or 15 ng/mL) to select the optimal dosage to stimulate HESCs. COL1A1, COL5A2 and FN expression in the different concentration groups is shown in Figure 2A. The expression of these proteins was TGF‐β1 concentration‐dependent, showing a peak of expression at 10 ng/mL. The mRNA expression also increased according to the TGF‐β1 concentration, as shown in Figure 2B. COL1A1, COL5A2 and FN mRNA expression increased in a dose‐dependent manner in response to TGF‐β1 treatment at 1‐10 ng/mL, and the differences between groups were statistically significant (*P* < .05). However, COL1A1, COL5A2 and FN mRNA expression did not differ between the 10 ng/mL TGF ‐β1 group and the 15 ng/mL TGF ‐β1 group. The optimal concentration of TGF ‐β1 for stimulating HESCs to fibrosis was 10 ng/mL, which was in accordance with our previous study.^19^ Thus, we chose 10 ng/mL TGF‐β1 to establish a cell fibrosis model for subsequent experiments.

### 3.2 Foxf2 down‐regulation or (and) Smad6 up‐regulation have anti‐fibrotic effects in TGF‐β1‐stimulated HESCs

Foxf2 and Smad6 may be important for the pathogenesis of IUA, as suggested by our previous study. Here, we showed that Foxf2 was up‐regulated and Smad6 was down‐regulated in the endometrium of patients with IUA (Figure 1B), suggesting that Foxf2 promoted fibrosis and Smad6 inhibited fibrosis. In the present study, we used si‐Foxf2 to down‐regulate Foxf2 expression and pcDNA3.1‐Smad6 to up‐regulate Smad6 expression in HESCs treated with 10 ng/mL TGF‐β1 to stimulate fibrosis. qRT‐PCR, Western blotting and IF were performed to examine the expression of Foxf2, Smad6, COL5A2, COL1A1, FN and α‐SMA.

#### 3.2.1 The mRNA expression of Foxf2, Smad6, COL5A2 and COL1A1 in each group

**Figure 3:**
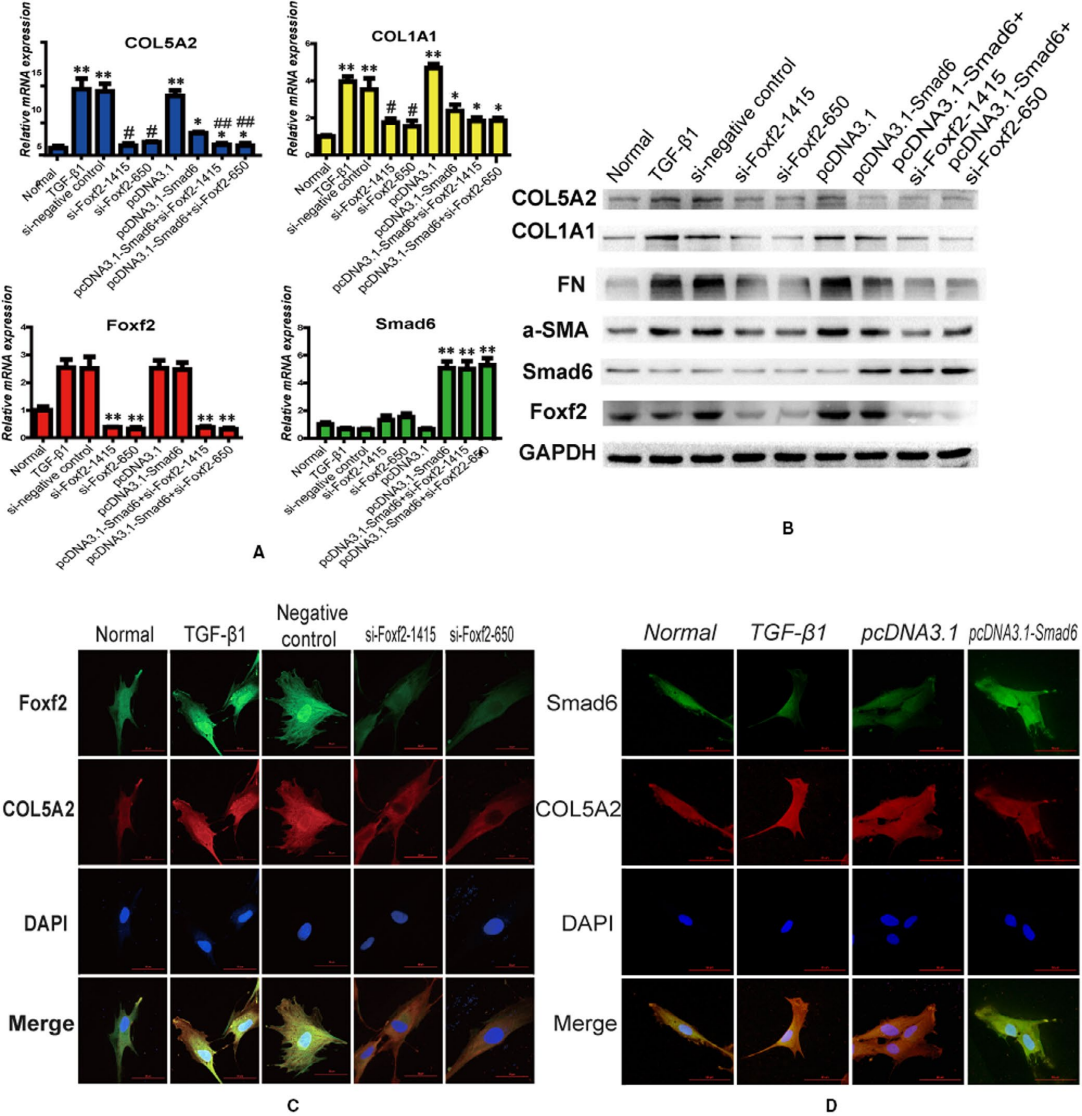
Foxf2 down‐regulation or (and) Smad6 up‐regulation have an anti‐fibrotic effects in TGF‐β1 stimulated cell fibrosis. HESCs were transfected with si‐Foxf2 or (and) pcDNA3.1‐Smad6 and then treated with TGF‐β1 (n = 3). (A) qRT‐PCR analysis of the COL5A2, COL1A1, Foxf2 and Smad6 expression in each group. Foxf2 and Smad6 mRNA expressions were obviously down‐regulated and up‐regulated by si‐Foxf2 and pcDNA3.1‐Smad6, respectively. The result showed that TGF‐β1 induced fibrosis, the expressions of COL5A2 and COL1A1 were increased by TGF‐β1, whereas down‐regulation of Foxf2 or (and) up‐regulation of Smad6 inhibited the expressions of COL5A2 and COL1A1 induced by TGF‐β1. **P < .05, compared with normal group. *P < .05, compared with pcDNA3.1 group. ^#^ *P* < .05, compared with TGF‐β1 and si‐negative control groups. ^##^ *P* < .05, compared with pcDNA3.1‐Smad6 group. (B) Western blotting analysis of the COL5A2, COL1A1, FN, a‐SMA, Foxf2 and Smad6 expressions in each group. (C) IF analysis of Foxf2 and COL5A2 expressions. The result showed that TGF‐β1 promoted COL5A2 expression, whereas down‐regulation of Foxf2 decreased COL5A2 expression induced by TGF‐β1. (D) IF analysis of Smad6 and COL5A2 expression. The result showed that TGF‐β1 promoted COL5A2 expression, whereas up‐regulation of Smad6 decreased COL5A2 expression induced by TGF‐β1

Transfection of cells with si‐Foxf2 decreased Foxf2 mRNA expression. The expressions of Foxf2 mRNA in the si‐Foxf2‐1415, si‐Foxf2‐650, pcDNA3.1‐Smad6+ si‐Foxf2‐1415 and pcDNA3.1‐Smad6+ si‐Foxf2‐650 groups were significantly lower than that in the normal group (*P* < .05). pcDNA3.1‐Smad6 up‐regulated Smad6 mRNA expression significantly, and the Smad6 mRNA expressions in the pcDNA3.1‐Smad6, pcDNA3.1‐Smad6+ si‐Foxf2‐1415 and pcDNA3.1‐Smad6+ si‐Foxf2‐650 groups were higher than that in the normal group (*P* < .05). These results indicated that both si‐Foxf2 and pcDNA3.1 effectively regulated the corresponding mRNA expression. TGF‐β1 played an important role in the pathogenesis of HESCs fibrosis, and the mRNA expression of COL5A2 and COL1A1 in the TGF‐β1, si‐negative control and pcDNA3.1 groups was higher than that in the normal group (*P* < .05), whereas COL5A2 and COL1A1 expression in the si‐Foxf2‐1415 and si‐Foxf2‐650 groups was significantly lower than that in the TGF‐β1 and si‐negative control groups (*P* < .05). Overexpression of Smad6 inhibited the expression of COL5A2 and COL1A1. The expressions of COL5A2 and COL1A1 in the pcDNA3.1‐Smad6 group were lower than those in the pcDNA3.1 group (*P* < .05). Cotransfection with si‐Foxf2 and pcDNA3.1‐Smad6 more efficiently inhibited COL5A2 expression than pcDNA3.1‐Smad6 alone, and the difference was statistically significant (*P* < .05). COL1A1 expression in the pcDNA3.1‐Smad6+ si‐Foxf2‐1415 and pcDNA3.1‐Smad6+ si‐Foxf2‐650 groups was lower than that in the pcDNA3.1‐Smad6 group, but the difference did not reach statistical significance (*P* > .05) (Figure 3A).

#### 3.2.2 Protein expression of Foxf2, Smad6, COL5A2, COL1A1, FN and α‐SMA in each group

The results of Western blot analysis of the protein expression of Foxf2, Smad6, COL5A2, COL1A1, FN and α‐SMA in each group are shown in Figure 3B. COL5A2, COL1A1, FN and a‐SMA were high expression in the TGF‐β1, si‐negative control and pcDNA3.1 groups, whereas they were low expression in the normal group. Down‐regulation of Foxf2 or up‐regulation of Smad6 inhibited protein expression induced by TGF‐β1. COL5A2, COL1A1, FN and α‐SMA expression was lower in the si‐Foxf2‐1415 and si‐Foxf2‐650 groups than in the TGF‐β1 and si‐negative control groups. COL5A2, COL1A1, FN and α‐SMA expression was lower in the pcDNA3.1‐Smad6 group than in the pcDNA3.1 group. COL5A2, COL1A1, FN and α‐SMA expression was lower in the si‐Foxf2‐1415+ pcDNA3.1‐Smad6 and si‐Foxf2‐650+ pcDNA3.1‐Smad6 groups than in the pcDNA3.1‐Smad6 group. The protein expression patterns were consistent with the respective mRNA expression patterns. The results of IF to evaluate COL5A2 expression after si‐Foxf2 or pcDNA3.1‐Smad6 transfection showed that COL5A2 expression was high in response to TGF‐β1, whereas it was reduced after transfection with si‐Foxf2 (Figure 3C). COL5A2 expression was decreased in response to Smad6 overexpression in TGF‐β1‐treated HESCs (Figure 3D).

These results indicated that Foxf2 down‐regulation or Smad6 up‐regulation inhibited COL5A2 and COL1A1 expressions in a HESC fibrosis model. Furthermore, cotransfection with si‐Foxf2 and pcDNA3.1‐Smad6 was more efficient for decreasing COL5A2 expression than pcDNA3.1‐Smad6 alone.

### 3.3 Foxf2 down‐regulation or/and Smad6 up‐regulation affects cell proliferation and cell cycle distribution induced by TGF‐β1

**Figure 4:**
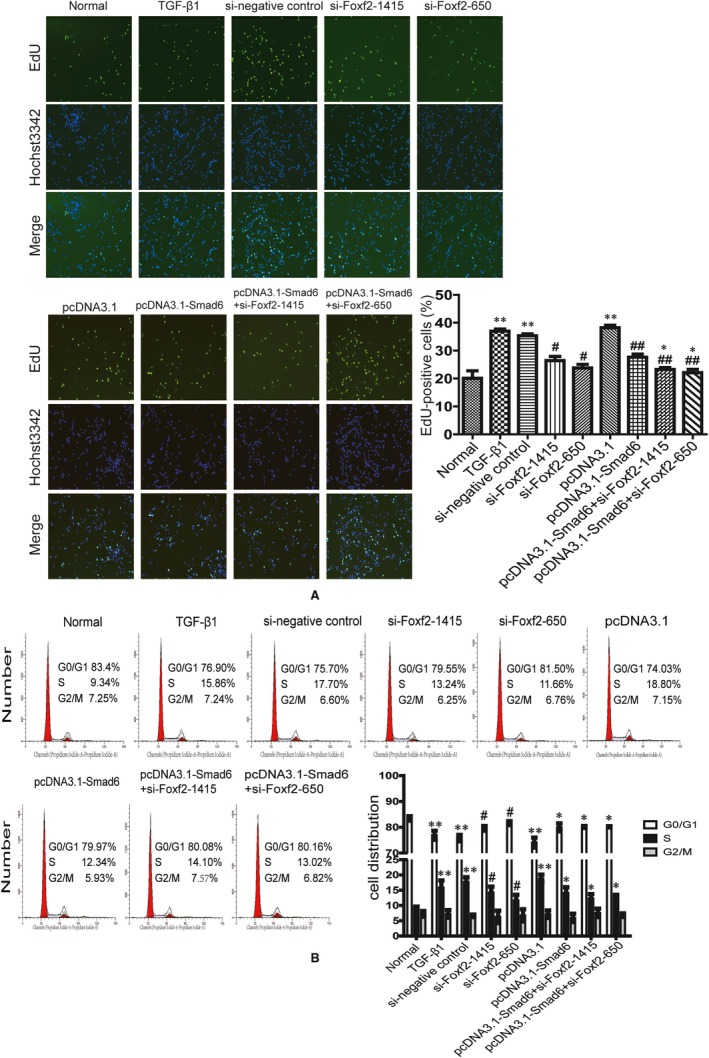
Foxf2 down‐regulation or/and Smad6 up‐regulation affects cell proliferation and cell cycle distribution induced by TGF‐β1 (n = 3). HESCs were transfected with si‐Foxf2 or (and) pcDNA3.1‐Smad6 and then treated with TGF β1 (A) EdU analysis of cell proliferation in each group. The result showed that TGF‐β1 increased EdU‐positive cell number, whereas down‐regulation of Foxf2 or (and) up‐regulation of Smad6 decreased EdU‐positive cell number induced by TGF‐β1. ** *P* < .05, compared with normal group. ^#^ *P* < .05, compared with TGF‐β1 and negative control groups. ^##^ *P* < .05, compared with pcDNA3.1 group. * *P* < .05, compared with pcDNA3.1‐Smad6 group. (B) Flow cytometry analysis of cell cycle distribution in each group. The result showed that TGF‐β1 promoted G0/G1 phase transition into S phase, whereas down‐regulation of Foxf2 or/and up‐regulation of Smad6 reversed the cell cycle changes induced by TGF‐β1. ** *P* < .05, compared with normal group. ^#^ *P* < .05, compared with TGF‐β1 and si‐negative control groups. * *P* < .05, compared with pcDNA3.1 group

We investigated the effect of Foxf2 down‐regulation or/and Smad6 up‐regulation on the proliferation of TGF‐β1‐stimulated HESCs using EdU essay (Figure 4A). The results showed that TGF‐β1 promoted cell proliferation; the EdU‐positive cell rate in TGF‐β1, si‐negative control and pcDNA3.1 groups was significantly higher than that in the normal group (*P* < .05). Down‐regulation of Foxf2 or up‐regulation of Smad6 expression significantly decreased the cell proliferation induced by TGF‐β1 compared with that in the TGF‐β1, si‐negative control and pcDNA3.1 groups (*P* < .05). Furthermore, cotransfection with si‐Foxf2 and pcDNA3.1‐Smad6 more effectively decreased cell proliferation than single transfection of pcDNA3.1‐Smad6 (*P* < .05).

Flow cytometry assessment of cell cycle distribution showed that stimulation with TGF‐β1 affected cell cycle distribution. TGF‐β1 activated HESCs and promoted G0/G1 phase transition into S phase, whereas Foxf2 down‐regulation or/and Smad6 up‐regulation reversed the cell cycle changes induced by TGF‐β1 (Figure 4B). The percentage of cells in S phase was significantly higher, whereas the percentage of cells in G0/G1 phase was significantly lower in the TGF‐β1, si‐negative control and pcDNA3.1 groups than in the normal group (*P* < .05). However, the percentage of cells in S phase was significantly lower, whereas the percentage of cells in G0/G1 phase was significantly higher in the si‐Foxf2‐1415 and si‐Foxf2‐650 groups than in the TGF‐β1 and si‐negative control groups (*P* < .05). The percentage of cells in S phase was significantly lower, whereas the percentage of cells in G0/G1 phase was significantly higher in the pcDNA3.1‐Smad6, pcDNA3.1‐Smad6+ si‐Foxf2‐1415 and pcDNA3.1‐Smad6+ si‐Foxf2‐650 groups than in the pcDNA3.1 group (*P* < .05). The percentage of cells in S phase was lower in the pcDNA3.1‐Smad6+ si‐Foxf2‐1415 and pcDNA3.1‐Smad6+ si‐Foxf2‐650 groups than in the pcDNA3.1‐Smad6 group, but the difference was not significant.

### 3.4 Foxf2 interacts with Smad6 and they co‐regulate COL5A2 transcription in the pathogenesis of HESC fibrosis

**Figure 5:**
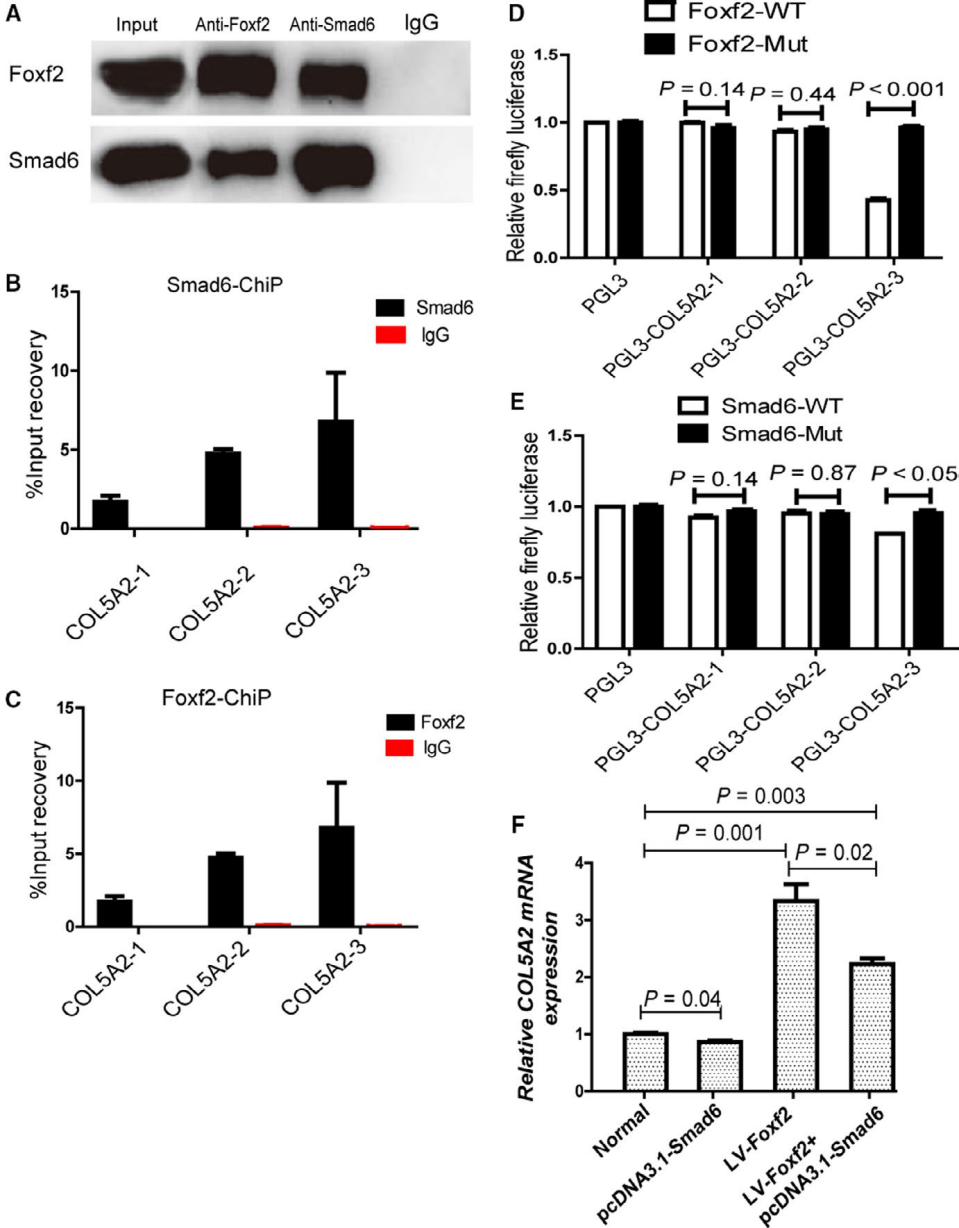
Foxf2 and Smad6 co‐regulate COL5A2 transcription in the pathogenesis of HESC fibrosis. (A) Co‐IP analysis of the interaction between Foxf2 and Smad6. Cell lysates were immunoprecipitated with anti‐Foxf2 antibody and anti‐Smad6 antibody, respectively, and were subjected to subsequent immunoblotting with anti‐Smad6 or anti‐FoxfF2 antibody. The result confirmed that Foxf2 interacts with Smad6. (B) ChIP analysis of the potential binding sites at the promoter region of COL5A2 that Smad6 bond. DNA fragments immunoprecipitated by anti‐Smad6 antibody were quantified by qPCR using primers covering predicted binding site. The result showed that Smad6 may bind at the promoter region of COL5A2. (C) ChIP analysis of the potential binding sites at the promoter region of COL5A2 that Foxf2 bond. DNA fragments immunoprecipitated by anti‐Foxf2 antibody were quantified by qPCR using primers covering predicted binding site. The result showed that Foxf2 may bind at the promoter region of COL5A2. (D) LV‐Foxf2 was cotransfected with PGL3, PGL3‐COL5A2‐1, PGL3‐COL5A2‐2, PGL3‐COL5A2‐3 into 293T cells (n = 3). Dual‐luciferase assay was performed to detect the activity and showed that Foxf2 bond PGL3‐COL5A2‐3. (E) pcDNA3.1‐Smad6 was cotransfected with PGL3, PGL3‐COL5A2‐1, PGL3‐COL5A2‐2, PGL3‐COL5A2‐3 into 293T cells. Dual‐luciferase assay was performed to detect the activity and showed that Smad6 bond PGL3‐COL5A2‐3. (F) RT‐PCR analysis of the effect of Foxf2 and Smad6 on COL5A2 expression. The result showed Foxf2 promoted COL5A2 expression, but Smad6 inhibited Foxf2‐induced COL5A2 expression

To determine whether Foxf2 interacts with Smad6 (http://genemania.org/), we performed Co‐IP in TGF‐β1‐stimulated HESCs by immunoprecipitation with anti‐Foxf2 antibody and immunoblotting with an anti‐Smad6 antibody. Conversely, lysates were immunoprecipitated with anti‐Smad6 antibody and immunoblotted with an anti‐Foxf2 antibody. The results showed that Foxf2 interacted with Smad6 in the pathogenesis of HESC fibrosis (Figure 5A).

Although both Foxf2 and Smad6 are related to the expression of COL5A2, the mechanism underlying the regulation of CO5A2 by Foxf2 and Smad6 remains unknown. We predicted the presence of binding sites for Foxf2 and Smad6 at the promoter region of COL5A2 and designed three binding site sequences (http://jaspar.genereg.net/, http://genome.ucsc.edu/). We performed ChIP assays to pull down Foxf2 or Smad6 bound DNA and qPCR to confirm. The results indicated that Foxf2 or Smad6 bound to the promoter region of COL5A2 to regulate its transcription (Figure 5B,C). Dual‐luciferase assays to identify the binding site for Foxf2 and Smad6 showed that both Foxf2 and Smad6 bound at chr2:190069038‐190069375 to regulate COL5A2 expression (Figure 5D,E). To evaluate the role of Foxf2 and Smad6 in COL5A2 transcription, LV‐Foxf2 and pcDNA3.1‐Smad6 were transfected into HESCs and qRT‐PCR was performed to examine COL5A2 expression. COL5A2 expression was higher in the LV‐Foxf2 group than in the normal group (*P* < .05), whereas COL5A2 expression was lower in the LV‐Foxf2+ pcDNA3.1‐Smad6 group than in the LV‐Foxf2 group (*P* ˂ .05) but higher than in the normal group (*P* < .05) (Figure 5F). These results indicated that Foxf2 promoted COL5A2 expression, and Smad6 reduced Foxf2‐induced COL5A2 expression without completely inhibiting it.

These results indicated that Foxf2 interact with Smad6 and bound to the same promoter region of COL5A2 to co‐regulate COL5A2 transcription in the pathogenesis of HESC fibrosis, whereas they had opposite effects on COL5A2 transcription.

### 3.5 Foxf2 down‐regulation or/and Smad6 up‐regulation decrease fibrosis in the pathogenesis of rat IUA

**Figure 6:**
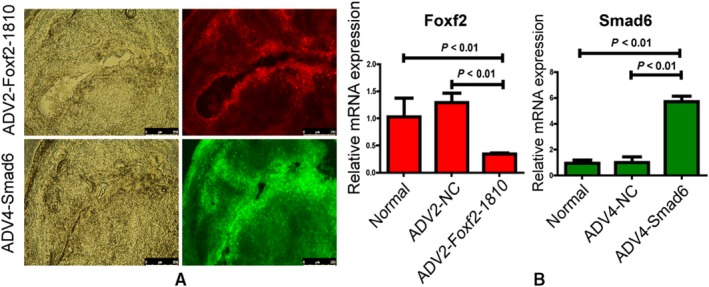
The efficiency of ADV2‐Foxf2 and ADV4‐Smad6 in rat endometrium. (A) Fluorescence analysis of uterine frozen section after transfected with ADV2‐Foxf2 or ADV4‐Smad6 (n = 3). The ADV2‐Foxf2‐1810 carried red fluorescence, and the ADV4‐Smad6 carried green fluorescence. (B) qRT‐PCR analysis of Foxf2 and Smad6 expression in rat endometrium after transfected with ADV2‐Foxf2‐1810 or ADV4‐Smad6. The red histogram represented relative Foxf2 mRNA expression, and ADV2‐FOXF2‐1810 decreased Foxf2 mRNA expression significantly compared with normal and ADV2‐negative control groups (*P* < .05). The green histogram represented relative Smad6 mRNA expression. ADV4‐Smad6 up‐regulated Smad6 mRNA expression significantly compared with normal and ADV4‐negative control groups (*P* < .05)

Foxf2 was down‐regulated using the ADV2 (CMV/IRES‐RFP) vector, and Smad6 was up‐regulated using the ADV4 (CMV/IRES‐RFP) vector. The efficiencies of transfection were verified by assessing fluorescence expression and qRT‐PCR as shown in Figure 6. The results demonstrated that ADV2‐Foxf2‐1810 significantly decreased Foxf2 mRNA expression and ADV4‐Smad6 significantly up‐regulated Smad6 mRNA expression in the female rat endometrium (*P* < .05).

#### 3.5.1 The number of endometrial glands in each group

**Figure 7:**
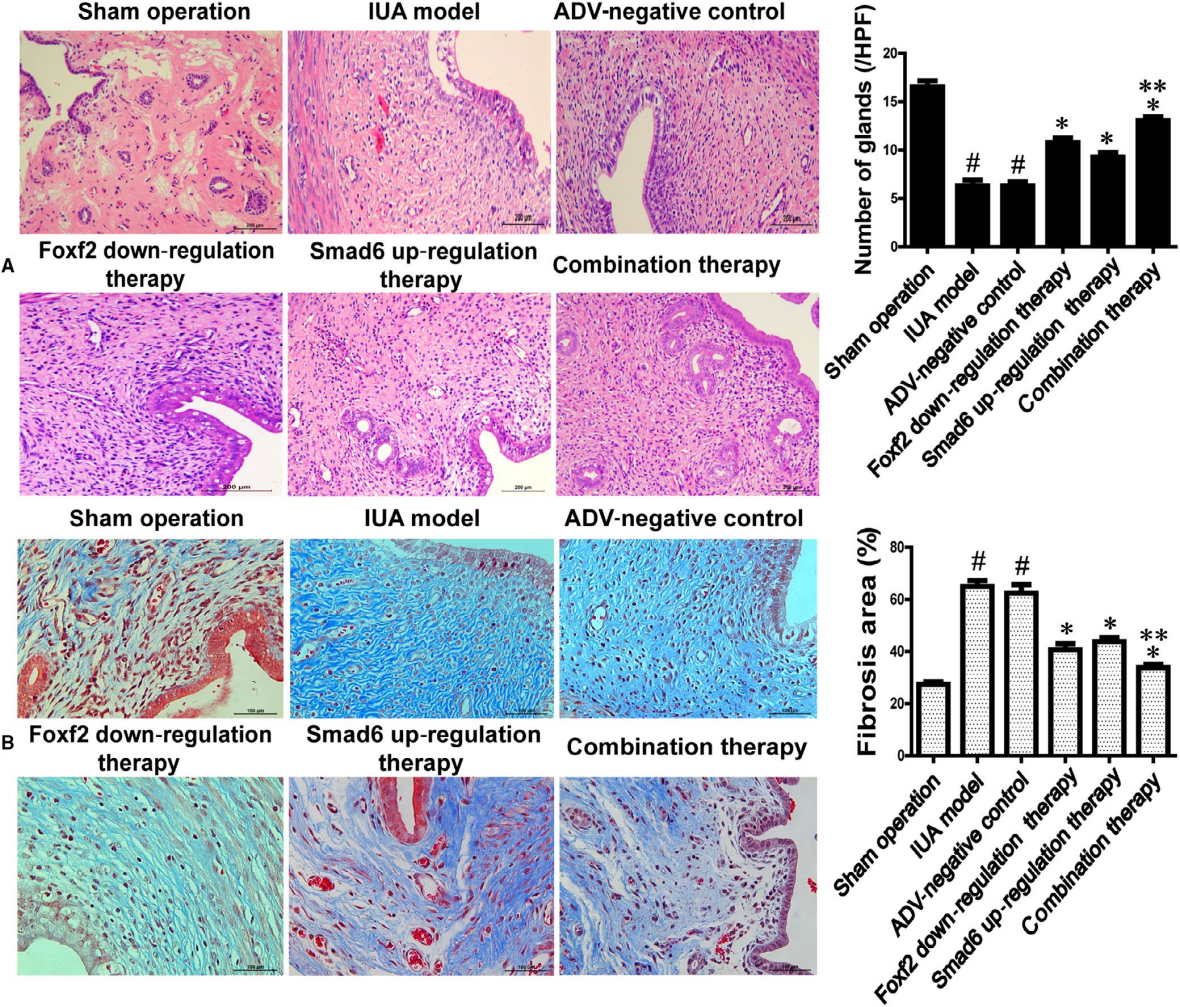
The effect of Foxf2 down‐regulation or (and) Smad6 up‐regulation on anti‐fibrosis in rat IUA model (n = 10). (A) HE staining analysis of the number of glands in rat endometrium in each group. ^#^ *P* < .05, compared with sham operation group. * *P* < .05, compared IUA model and ADV‐negative control group. ** *P* < .05, compared with Smad6 up‐regulation therapy group. (B) Masson staining of analysis of the percentage of fibrosis area in each group. ^#^ *P* < .05, compared with sham operation group. * *P* < .05, compared with IUA model and ADV‐negative control group. ** *P* < .05, compared with Smad6 up‐regulation therapy group

After transfection of the ADV vector in the IUA model, the uteri were collected and sectioned two weeks after IUA development. HE staining was performed to examine the glands in the endometrium, which showed normal endometrial morphology in the sham‐operated group, with round or oval glands distributed regularly in the submucosa and basal layer of the endometrium, and columnar epithelial cells covering the endometrial surface. However, the number of glands in the endometrium was substantially decreased in the IUA model group, indicating the successful generation of the IUA model. The average number of glands in the sham group was 16.50 ± 1.29, which was higher than that in the IUA model (6.25 ± 1.26) and ADV‐negative control group (6.25 ± 0.96) (*P* < .05). The average number of glands in the Foxf2 down‐regulation and Smad6 up‐regulation groups was 10.75 ± 1.01 and 9.25 ± 0.95, respectively, which was significantly higher than that in the IUA model or ADV‐negative control groups (*P* < .05). The average number of glands in the combination therapy group was 13.00 ± 0.90 which was higher than that in the Foxf2 down‐regulation and Smad6 up‐regulation groups, although the difference was only significant in the Smad6 up‐regulation group (Figure 7A).

#### 3.5.2 The degree of rat endometrial fibrosis in each group

Masson staining was used to evaluate the degree of fibrosis (Figure 7B). In the sham group, the fibrosis area was barely detectable, and the endometrial stroma rarely appeared blue, whereas the endometrial stroma in the IUA model and ADV‐negative control groups appeared dark blue. The percentage of fibrosis area in the IUA model and ADV‐negative control groups was 64.95% ± 4.76% and 62.37% ± 6.67%, respectively, which was significantly higher than that in the sham group (27.35% ± 1.88%) (*P* < .05). However, the percentage of fibrosis area in the Foxf2 down‐regulation group (40.75% ± 4.53%) and the Smad6 up‐regulation group (43.77% ± 3.01%) was significantly lower than that in the IUA model and ADV‐negative control groups (*P* < .05). The percentage of the fibrosis area in the combination therapy group was 33.77% ± 2.43%, which was lower than that in the Foxf2 down‐regulation group and Smad6 up‐regulation group, although the difference was only significant in the Smad6 up‐regulation group.

These results indicated that Foxf2 down‐regulation or Smad6 up‐regulation decreased fibrosis in association with the pathogenesis of rat IUA, and both together were more effective than each alone.

### 3.6 Foxf2 down‐regulation or/and Smad6 up‐regulation decreases collagen production in the pathogenesis of rat IUA

#### 3.6.1 The expression of Foxf2 and Smad6 in the rat endometrium of each group

**Figure 8:**
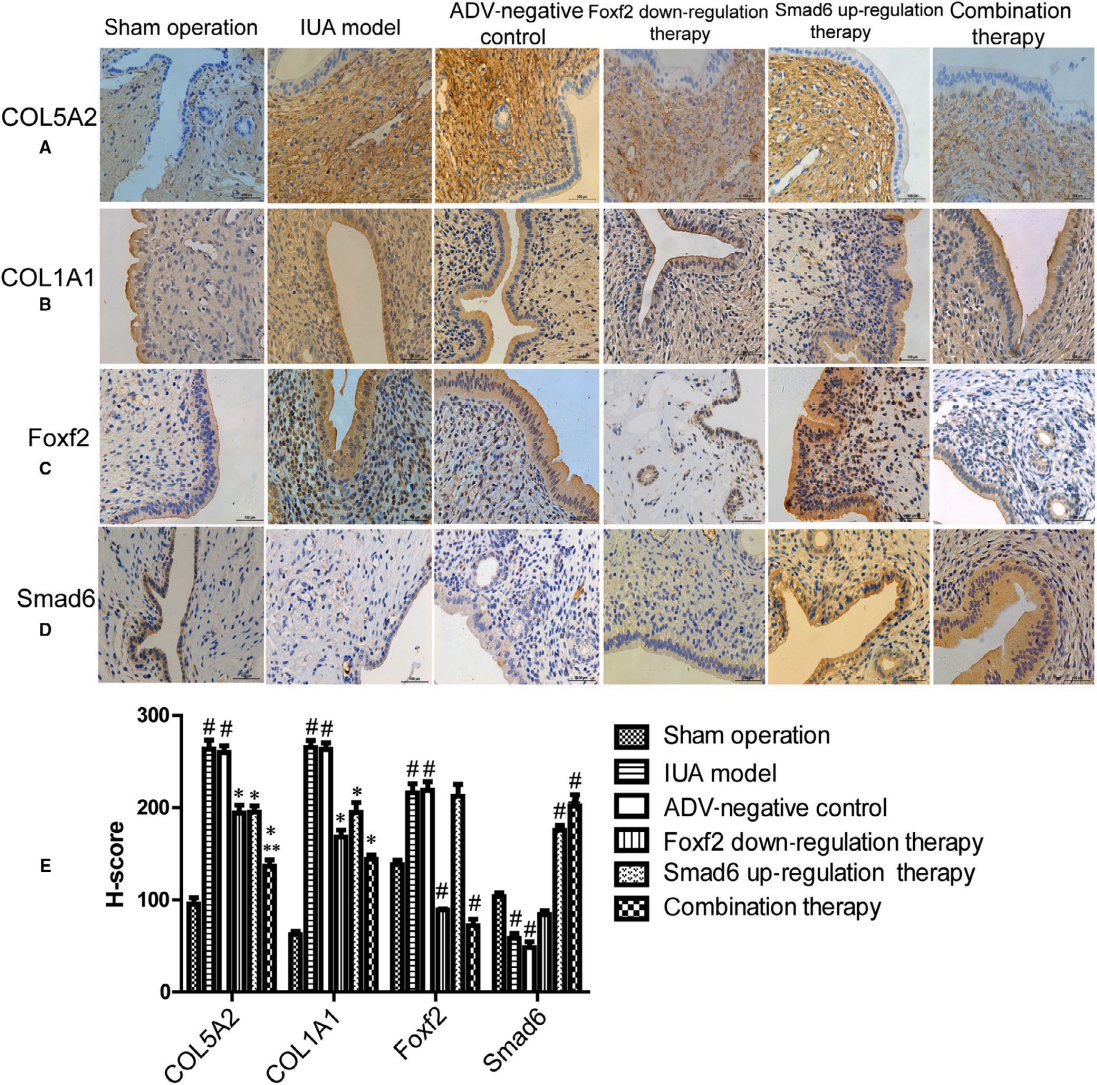
The effect of down‐regulation of Foxf2 or (and) up‐regulation of Smad6 on collagen productions in rat IUA model (n = 10). (A) HE immunostaining analysis of COL5A2 expression in each group. (B) HE immunostaining analysis of COL1A1 expression in each group. (C) HE immunostaining analysis of Foxf2 expression in each group. (D) HE immunostaining analysis of Smad6 expression in each group. (E)The immunostaining was evaluated by semi‐quantitatively scored using the modified histochemical score (H‐score), and data were showed as mean ± SD. The result showed that down‐regulation of Foxf2 or (and) up‐regulation of Smad6 inhibited COL1A1 and COL5A2 expression in rat IUA model. ^#^ *P* < .05, compared with sham operation group. * *P* < .05, compared IUA model and ADV‐negative control group. ** *P* < .05, compared with Smad6 up‐regulation therapy group

Foxf2 is a transcription factor that is synthesized in the cytoplasm and transported into the nucleus to activate its target genes. In the present study, Foxf2 expression was higher in the IUA model, ADV‐negative control and Smad6 up‐regulation therapy groups than that in the sham operation group (216 ± 17.1 vs 138.0 ± 8.5, *P* < .05 and 218.7 ± 16.4 vs 138.0 ± 8.5, *P* < .05 and 212.3 ± 22.3 vs 138.0 ± 8.5, *P* < .05, Figure 8C). Foxf2 expression was lower in the Foxf2 down‐regulation and combination therapy group than that in the sham operation group (89.0 ± 2.1 vs 138.0 ± 8.5, *P* < .05 and 72.0 ± 112.5 vs 138.0 ± 8.5, *P* < .05).

Smad6 is an inhibitory regulator of the TGF‐β/Smad signalling pathway involved in fibrosis. The expression of Smad6 in the IUA model and ADV‐negative control groups was significantly lower than in the sham operation group (58.3 ± 9.1 vs 103.6 ± 6.7, *P* < .05 and 48.3 ± 10.9 vs 103.6 ± 6.7, *P* < .05). And the expression of Smad6 in the Smad6 up‐regulation and combination therapy groups was higher than in the sham operation group (175.3 ± 9.3 vs 103.6 ± 6.7, *P* < .05 and 201.6 ± 21.1 vs 103.6 ± 6.7, *P* < .05) (Figure 8D).

These results indicated that ADV2‐Foxf2‐1810 down‐regulated Foxf2 protein expression, and ADV4‐Smad6 up‐regulated Smad6 protein in vivo. Moreover, Foxf2 was up‐regulated and Smad6 was down‐regulated in association with the pathogenesis of rat IUA.

#### 3.6.2 The expression of COL5A2 and COL1A1 in the rat endometrium of each group

COL5A2 and COL1A1 are expressed in the cytoplasm and stroma, and both show high expression in fibrosis. As shown in Figure 8A, the expression of COL5A2 in the IUA model and ADV‐negative control groups was significantly higher than in the sham operation group (263.3 ± 16.8 vs 95.3 ± 12.2, *P* < .01 and 265.0 ± 13.5 vs 95.3 ± 12.2, *P* < .01). COL5A2 expression was significantly lower in the Foxf2 down‐regulation, Smad6 up‐regulation and combination therapy groups than in the ADV‐negative control group (194.0 ± 15.1 vs 259.6 ± 12.9, *P* < .01 and 195.1 ± 11.8 vs 259.6 ± 12.9, *P* < .01 and 136.6 ± 12.5 vs 259.6 ± 12.9, *P* < .01). COL5A2 expression was significantly lower in the combination therapy group (136.6 ± 12.5) than in the Smad6 up‐regulation and Foxf2 down‐regulation groups (*P* < .05).

The COL1A1 expression pattern was similar to that of COL5A2 (Figure 8B). COL1A1 expression was significantly higher in the IUA model and ADV‐negative control groups than in the sham operation group (265.0 ± 13.5 vs 62.3 ± 6.1, *P* < .01 and 263.0 ± 12.1 vs 62.3 ± 6.1, *P* < .01). COL1A1 expression was significantly lower in the Foxf2 down‐regulation, Smad6 up‐regulation and combination therapy groups than in the ADV‐negative control group (168.0 ± 13.1 vs 263.0 ± 12.1, *P* < .01 and 194.7 ± 19.2 *P* < .01 and 114.0 ± 2.2 vs 263.0 ± 12.1, *P* < .01). COL1A1 was lower in the combination therapy group than in the Foxf2 down‐regulation and Smad6 up‐regulation groups, but the difference was not significant.

These results indicated that Foxf2 down‐regulation and Smad6 up‐regulation inhibited COL5A2 and COL1A1 expression in association with the pathogenesis of rat IUA.

## 4 DISCUSSION

Intrauterine adhesion is a major reproductive problem for childbearing age women, and it can lead to menstrual abnormalities, pelvic pain, recurrent abortion, infertility and pregnancy‐related complications.^1^ The incidence of IUA following early pregnancy loss is 6.3%.^28^ Moreover, 36‐53 million pregnancies are terminated every year worldwide, of which approximately 90% are terminated in the first trimester.^29^ In the United States, approximately 1.2 million abortions were performed in 2008.^30^ Therefore, IUA remains a public health problem for premenopausal women that needs attention. Clarifying the mechanism underlying the pathogenesis of IUA is important. Dilatation and curettage (D&C), a primary risk factor for IUA, causes damage to the basal layer of the endometrium leading to endometrial fibrosis, in which fibrous tissue replaces stromal tissue accompanied with a decrease or disappearance of glands. As a result, the uterine cavity and/or the cervical canal become partially or completely obliterated.^31^ IUA is characterized by the replacement of the normal endometrium by fibrous tissue as a result of injury to the basal layer; therefore, uncovering the mechanism underlying fibrous tissue formation is important. Collagen, which is the main constituent of fibrous tissue, is widely and abundantly expressed in fibrous tissue.^10^ In our previous study, we showed that the expression of COL5A1 and COL1A1 is higher in the IUA endometrium than in the normal endometrium, and COL5A2 expression is positively associated with the degree of IUA (Figure 1B).

COL5A2 is a subtype of collagen V that is defined as regulatory fibril‐forming collagen. Collagen fibrils are often heterogeneous and contain more than one collagen type^32^. Collagen V plays an important role in the formation of fibrils and combined with collagens I, III and XII; it forms fibrils that are deposited in the ECM, with collagen V localizing to the core of fibrils.^11^ COL5A2 is involved in the pathogenesis of fibrosis in vivo and in vitro. In COL5A2^−/−^ mice, lack of expression of COL5A2 leads to disorganized type I collagen fibrils, and mice exhibit eye and skin abnormalities.^30^ The expression of COL5A2 is significantly up‐regulated in association with fibrosis in renal epithelial cell lines and rat liver fibrosis.^13^, ^33^ In our previous study, we demonstrated that COL5A2 expression is up‐regulated and correlated with the degree of IUA (Figure 1). In the present study, we confirmed that COL5A2 is overexpressed in HESCs with fibrosis and in a rat IUA model (Figures 3 and 8B). This suggested that COL5A2 plays a vital role in the pathogenesis of IUA. It is important to explore the mechanism regulating COL5A2 expression to improve our understanding of the development of IUA. However, the mechanism underlying the regulation of COL5A2 production and its association with the pathogenesis of IUA remain unclear.

Foxf2 is a member of the Fox family of transcription factors that play a vital role in cell growth and tissue development and is ubiquitously expressed in mesenchymal cells.^34^ It is located on chromosome 6p25.3 and has a “winged helix” DNA binding domain characterized by a highly conserved sequence that mediates its interaction with target genes such TATA‐box binding protein (TBP) and transcription factor TFIIB (TFIIB) to promote or inhibit transcription.^35^ Foxf2 is important for ECM formation in intestinal fibrosis. In Foxf2**^−/−^** mice, the collagen in the intestine is strikingly deficient, and cell adhesion is defective.^16^ In the present study, Foxf2 was significantly overexpressed in vivo and in vitro in fibrotic cells (Figures 3B and 8C). The results indicated that Foxf2 promoted collagen production in association with the pathogenesis of fibrosis. This was consistent with previous reports. Zhu et al reported that inhibition of FoxF2 expression increases mesenchymal Wnt5a expression, which activates the canonical Wnt signalling pathway resulting in epithelial depolarization and tissue disintegration.^36^ Other studies also reported that mesenchymal tissues are substantially decreased in Foxf2^−/−^ palatal shelf.^37^

The TGF‐β family plays important roles in embryonic development and fibrosis. TGF‐β induces collagen production in various renal cells, such as glomerular mesangial cells, tubular epithelial cells and renal fibroblasts.^38^, ^39^ In the present study, we used TGF‐β1 to establish a cell fibrosis model, and the results showed that TGF‐β1 induced the expression of COL5A2, COL1A1, a‐SMA and FN in HESCs (Figures 2 and 3A). Smads are signalling mediators of the TGF‐β superfamily, and there are three classes of Smads: (a) regulatory Smads (R‐Smads), including Smad1, Smad2, Smad3, Smad5 and Smad8; (b) inhibitory Smads, including Smad6 and Smad7; and (c) common Smads, that is Smad4.^40^ In the TGF‐β family signalling pathway, R‐Smads are phosphorylated and form a heteromer with Smad4, which is transferred into the nucleus to activate target genes. However, Smad6 is a negative regulator that interferes with the phosphorylation of Smad1 and Smad2 and disturbs the formation of the heteromer.^41^ Smad6 has anti‐fibrotic effects in conjunctival fibroblasts and a mouse model of glaucoma filtration surgery, and overexpression of Smad6 attenuates TGF‐β‐induced collagen production.^22^ CCN5 is one of the connective tissue growth factor/cysteine‐rich 61/nephroblastoma overexpressed (CCN) family that has been shown to play important roles in many processes, including adhesion, extracellular matrix regulation, proliferation, migration. In epidural fibrosis, CCN5 exerts anti‐fibrotic effects by regulating the Smad6‐CCN2 pathway.^42^

Foxf2 and Smad6 play crucial roles in the production of collagen. Based on our previous study showing that Foxf2 is up‐regulated and Smad6 is down‐regulated in IUA patients compared with healthy controls (Figure 1), we have been suggested that Foxf2 and Smad6 are involved in the pathogenesis of IUA. To prove this hypothesis, we down‐regulated Foxf2 or (and) up‐regulated Smad6 expression in a TGF‐β1‐induced HESC fibrosis model. Down‐regulation of Foxf2 and up‐regulation of Smad6 decreased COL1A1, COL5A2, a‐SMA and FN expression in association with HESC fibrosis. The combination of Foxf2 down‐regulation and Smad6 up‐regulation was more effective at reducing COL5A2 expression than single treatment (Figure 3A,B). In vivo, IHC experiments showed that Foxf2 was up‐regulated and Smad6 was down‐regulated in the rat IUA endometrium. These results indicated that Foxf2 and Smad6 play important roles in the pathogenesis of IUA.

We then examined the effect of Foxf2 and Smad6 on cell proliferation and cell cycle distribution. TGF‐β controls many fundamental cell behaviours, stimulating proliferation and altering cell cycle distribution.^43^ In the present study, TGF‐β1 induced HESC proliferation and promoted cell cycle progression from G0/G1 to S phase (Figure 4A,B). However, it had been reported that TGF‐β1 inhibited cell proliferation in many studies.^44^, ^45^, ^46^ TGF‐β1 may have a dual role on cell proliferation, and there had also many studies reported that TGF‐β1 promotes proliferation.^47^, ^48^

The TGF‐β signalling pathway is initiated when an activated TGF‐β ligand binds to its membrane receptors, TGF‐β type II receptor (TβRII) and TGF‐β type I receptor (TβRI). This results in the formation of a complex that activates R‐Smad phosphorylation and combines with Smad4 to exert its biological activities.^19^ Smad6 competes with R‐Smads and inhibits R‐Smad phosphorylation and activation to change cell behaviours.^20^ Other transcription factors can regulate TGF‐β1 signalling. It had been reported that forkhead subfamily proteins interact with Smad3‐Smad4 complexes in epithelial cells and promote the expression of the CDK inhibitors p21^CIP1^ and p15^INK4B^, affecting cell cycle progression.^49^ In the present study, both down‐regulation of Foxf2 and up‐regulation of Smad6 inhibited TGF‐β1‐induced proliferation and cell cycle distribution.

Foxf2 and Smad6 are transcription factors and play important roles in the fibrosis of HESCs, although the underlying mechanism remains unclear. Smad3 and Smad4 interact with the forkhead transcription factor FOXL2 to regulate Fshb, Gnrhr and Fst transcription in vitro.^50^ Whether Foxf2 interacts with Smad6 during fibrosis development has not been reported to date. We performed Co‐IP assays to detect whether Foxf2 interacted with Smad6 in HESC fibrosis. As shown in Figure 5A, Foxf2 interacted with Smad6 in association with the pathogenesis of HESC fibrosis.

In the pathogenesis of HESC fibrosis, Foxf2 and Smad6 interact with each other and play opposite roles in collagen production, although the underlying mechanism remains unclear. Based on the finding that combined down‐regulation of Foxf2 and up‐regulation of Smad6 reduced COL5A2 expression more effectively than single treatment (Figure 3A,B), we predicted that Foxf2 and Smad6 may bind to the promoter region of COL5A2 and designed three binding site sequences to which Foxf2 and Smad6 could bind (http://jaspar.genereg.net/, http://genome.ucsc.edu/). ChIP and luciferase assays were performed to test the interaction. The results of ChIP assay showed that there are potential binding sites to which Foxf2 and Smad6 may bind at the promoter region of COL5A2 (Figure 5B,C). We then constructed a vector and performed luciferase assays to determine which binding site they bind to. The results showed that Foxf2 and Smad6 bind at chr2:190069038‐190069375 to regulate COL5A2 transcription (Figure 5D,E). Foxf2 and Smad6 bind to the same promoter region of COL5A2 to regulate its transcription in fibrotic HESCs. For further understanding the role of Foxf2 and Smad6 in COL5A2 expression, we transfected LV‐Foxf2 and pcDNA3.1‐Smad6 into HESCs and qRT‐PCR was performed to detect COL5A2 expression. The results showed that Foxf2 promoted COL5A2 expression and Smad6 inhibited Foxf2‐induced COL5A2 expression. Therefore, we concluded that Foxf2 and Smad6 had an opposite role in the pathogenesis of fibrosis.

In the present study, we showed that Foxf2 and Smad6 are involved in the pathogenesis of fibrosis in vitro and in vivo. Foxf2 promoted collagen production, whereas Smad6 inhibited it, and both factors bound to the same promoter region of COL5A2 to regulate its transcription. However, the balance between Foxf2 and Smad6 for regulating COL5A2 remains unclear. The regulation of COL5A2 transcription by Foxf2 and Smad6 may involve competition between the two factors for binding to the promoter region of COL5A2, or Smad6 interaction with Foxf2 to change its activity in binding to the promoter region of COL5A2.

## 5 CONCLUSION

The present results suggested that Foxf2 and Smad6 co‐regulated COL5A2 transcription in the pathogenesis of IUA. Foxf2 down‐regulation or/and Smad6 up‐regulation may inhibit collagen production and cell proliferation during fibrosis, providing a potential new strategy for the prevention and treatment of IUA.

## CONFLICT OF INTEREST

The author(s) declared no potential conflicts of interest concerning the research, authorship and publication of this article.

## AUTHORS' CONTRIBUTIONS

Yuanli He, Guobin Chen and Huihua Cai designed the experiments. Guobin Chen, Limin Liu, Jing Sun and Huihua Cai performed experiments. Guobin Chen conducted statistical analyses. Guobin Chen wrote the first draft of the manuscript, and all authors commented on the subsequent draft. Yuanli He reviewed the final draft. This work was done in the Central Laboratory of Zhujiang Hospital, Southern Medical University and Research Center of Clinical Medicine of Nanfang Hospital, Southern Medical University.
